# Disparity in the Influence of Implant Provisional Materials on Human Gingival Fibroblasts with Different Phases of Cell Settlement: An In Vitro Study

**DOI:** 10.3390/ijms25010123

**Published:** 2023-12-21

**Authors:** Takanori Matsuura, Stella Stavrou, Keiji Komatsu, James Cheng, Alisa Pham, Stephany Ferreira, Tomomi Baba, Ting-Ling Chang, Denny Chao, Takahiro Ogawa

**Affiliations:** 1 Weintraub Center for Reconstructive Biotechnology, Division of Regenerative and Reconstructive Sciences, UCLA School of Dentistry, Los Angeles, CA 90095, USA; stella.c.stavrou@gmail.com (S.S.); jamescheng@dentistry.ucla.edu (J.C.); alisapham@dentistry.ucla.edu (A.P.); uclaosugi@gmail.com (T.B.); tlchang@dentistry.ucla.edu (T.-L.C.); denchao@ucla.edu (D.C.); togawa@dentistry.ucla.edu (T.O.); 2 West Los Angeles VA Medical Center, Los Angeles, CA 90073, USA; stephanyferreira3@gmail.com

**Keywords:** implant provisional materials, peri-implant soft tissue, cytotoxicity, cytocompatibility, gingival fibroblasts

## Abstract

The development of healthy peri-implant soft tissues is critical to achieving the esthetic and biological success of implant restorations throughout all stages of healing and tissue maturation, starting with provisionalization. The purpose of this study was to investigate the effects of eight different implant provisional materials on human gingival fibroblasts at various stages of cell settlement by examining initial cell attachment, growth, and function. Eight different specimens—bis-acrylic 1 and 2, flowable and bulk–fill composites, self-curing acrylic 1 and 2, milled acrylic, and titanium (Ti) alloy as a control—were fabricated in rectangular plates (*n* = 3). The condition of human gingival fibroblasts was divided into two groups: those in direct contact with test materials (contact experiment) and those in close proximity to test materials (proximity experiment). The proximity experiment was further divided into three phases: pre-settlement, early settlement, and late settlement. A cell culture insert containing each test plate was placed into a well where the cells were pre-cultured. The number of attached cells, cell proliferation, resistance to detachment, and collagen production were evaluated. In the contact experiment, bis-acrylics and composites showed detrimental effects on cells. The number of cells attached to milled acrylic and self-curing acrylic was relatively high, being approximately 70% and 20–30%, respectively, of that on Ti alloy. There was a significant difference between self-curing acrylic 1 and 2, even with the same curing modality. The cell retention ability also varied considerably among the materials. Although the detrimental effects were mitigated in the proximity experiment compared to the contact experiment, adverse effects on cell growth and collagen production remained significant during all phases of cell settlement for bis-acrylics and flowable composite. Specifically, the early settlement phase was not sufficient to significantly mitigate the material cytotoxicity. The flowable composite was consistently more cytotoxic than the bulk–fill composite. The harmful effects of the provisional materials on gingival fibroblasts vary considerably depending on the curing modality and compositions. Pre-settlement of cells mitigated the harmful effects, implying the susceptibility to material toxicity varies depending on the progress of wound healing and tissue condition. However, cell pre-settlement was not sufficient to fully restore the fibroblastic function to the normal level. Particularly, the adverse effects of bis-acrylics and flowable composite remained significant. Milled and self-curing acrylic exhibited excellent and acceptable biocompatibility, respectively, compared to other materials.

## 1. Introduction

In implant therapy, a soft tissue seal is crucial for preventing bacterial invasion and maintaining the long-term health of peri-implant tissues [1,2,3,4,5]. Peri-implant soft tissue heals and develops during various stages of provisionalization, including implant surgery, second-stage surgery, subsequent wound healing, emergence profile formation, and tissue maturation [6,7,8,9]. Implant provisional restorations are typically made of polymer-based materials. Depending on the modality of cure and chemical composition, these resins can be classified as self-cured, light-cured, or heat-cured acrylic or composite resins.

The biological properties and responses of the peri-implant soft tissues can be influenced by the chemical composition of provisional restorative materials [10,11,12,13]. Some studies have reported that acrylic materials are cytotoxic mainly due to the continued release of residual monomers even after polymerization [14,15,16,17,18,19]. In chemical cured materials, almost all materials are mainly polymethyl methacrylate (PMMA)-based or bis-acrylic-based. PMMA is generated by the polymerization of MMA, and it has high moldability and tractability [20,21,22,23]. Some bis-acrylics contain fillers to prevent curing shrinkage and photoinitiators to improve handling and reduce curing time [24,25,26,27]. PMMA disks are chemically pre-polymerized under high-temperature and high-pressure conditions. These disks can be milled into the desired shape using computer-aided design/computer-aided manufacturing (CAD/CAM) systems. Prefabricated PMMA disks are assumed to produce minimal or no residual monomers or free radicals [28,29,30,31]. In light-cured materials, composites with high fluidity contain fewer fillers and lower viscosity matrix materials, such as triethylene glycol dimethacrylate (TEGDMA), while composites with lower fluidity contain more fillers and higher viscosity matrix materials, such as urethane dimethacrylate (UDMA) [32,33,34]. In many studies, the addition of eluates from acrylic materials to culture medium is commonly performed [17,35,36,37,38,39]. This method allows for maintaining consistent concentration; however, the actual components leaching from the materials tend to decrease over time. Therefore, by conducting cell culture in contact and proximity to the actual material itself, a more accurate evaluation of cellular responses to an in vivo environment is possible.

Fibroblasts play a key role in wound healing by contributing to formation of the extracellular matrix (ECM) components such as collagen, glycoproteins, and other constituents of the developed peri-implant soft tissue, creating a soft tissue seal in the connective tissue [40,41,42]. To maintain tissue function, cells adhere, settle, and interact with other cells and the extracellular matrix in order to maintain the structure and organization of tissues through these complex cellular adhesion mechanisms [43,44]. The influence of external factors, such as restorative materials, may vary depending on the condition of cell settlement [45]. In our previous in vitro studies, the direct contact and close proximity effects of five different provisional materials on fibroblasts and osteoblasts were assessed [46,47]. The cells were seeded simultaneously with the placement of materials; thus, the cell settlement conditions were not taken into consideration. Peri-implant soft tissue heals and develops during various stages of provisionalization, including implant surgery, second-stage surgery, subsequent wound healing, emergence profile formation, and tissue maturation. We need to investigate the cell response to materials using a systemic experimental model that considers these tissue healing processes.

Therefore, the objective of this study is to investigate the effects of eight different provisional materials, including titanium (Ti) alloy as a control, on human gingival fibroblasts at various stages of cell settlement by examining initial cell attachment, growth, and function. We hypothesized that there is a substantial difference between materials before cell settlement, but the differences decrease as cells settle.

## 2. Results

### 2.1. Growth of Fibroblasts on Test Materials

**Figure 1:**
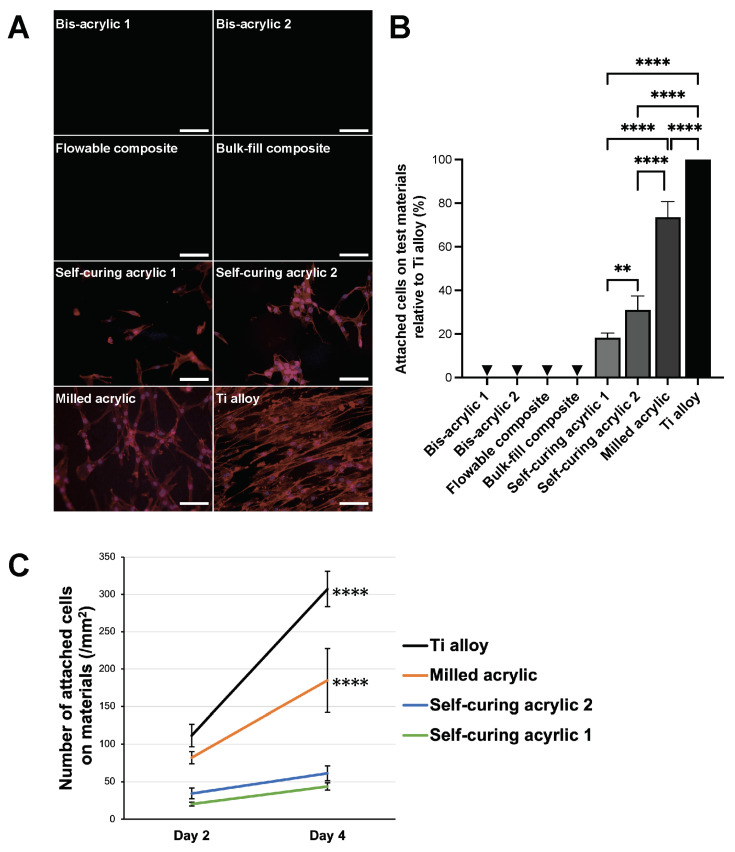
Fibroblasts growth on test materials. (**A**) Visualization of fibroblasts on test materials 2 days after seeding. Fibroblasts were dual stained with fluorescent dyes, DAPI for nuclei, and rhodamine–phalloidin for actin filaments. Scale bars indicate 1 mm. (**B**) Percentage of the number of cells attached to the test materials relative to the number of cells on Ti alloy was measured. Arrowheads indicate not applicable. One-way ANOVA, followed by Tukey–Kramer post hoc test, *p* ** < 0.01, *p* **** < 0.0001. (**C**) Cell propagation from day 2 to day 4. Data shown are means ± SD. Two-way ANOVA, followed by the Fisher’s LSD post hoc test. *p* **** < 0.0001.

To evaluate the success of cell settlement to test plates, fibroblasts on the plates were visualized by fluorescent microscopy, and the number of attached cells was quantified (Figure 1A). Cell nuclei were stained with DAPI, and actin filaments were stained with rhodamine. Fibroblasts on Ti alloy exhibited a spindle shape and were aligned in the same direction. The cells on milled acrylic were spindle-shaped and spread randomly. A few small fibroblasts were attached to self-curing acrylics. No cells were attached to any other materials.

Cell numbers were determined by counting cell nuclei, and the percentage of cells attached to each test material was calculated relative to that of Ti alloy (Figure 1B). The percentage for milled acrylic was 73.52 ± 7.25% of that for Ti alloy. Irrespective of the same category, the percentage for self-curing acrylic 2 was approximately two times higher than that of self-curing acrylic 1 (31.00 ± 6.44% and 18.21 ± 2.26%, respectively) (*p* = 0.042). To assess cell proliferation, changes in cell numbers from day 2 to day 4 were evaluated (Figure 1C). On day 4, cell numbers for Ti alloy and milled acrylic were more than two times higher than on day 2 (307.33 ± 23.62 cells/mm^2^ vs. 111.43 ± 14.79 cells/mm^2^, and 184.83 ± 42.67 cells/mm^2^ vs. 81.93 ± 8.08 cells/mm^2^, respectively) (*p* < 0.0001), while there were no significant increases for self-curing acrylic 1 and 2.

### 2.2. Cell Retention Ability

**Figure 2:**
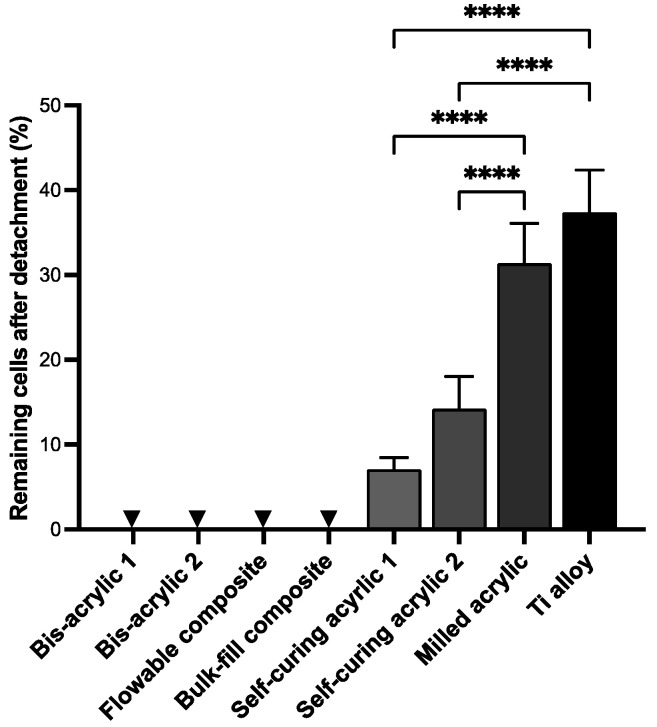
Cell retention on test materials. Cell retention was evaluated by calculating the percentage of remaining cells after chemical detachment. Arrowheads indicate not applicable. Data shown are means ± SD. One-way ANOVA followed by the Tukey–Kramer post hoc test. **** *p* < 0.0001.

Next, we assessed the retention of fibroblasts once attached to the materials. Fibroblasts attached to the materials for four days were subjected to chemical detachment, and the percentage of remaining cells was calculated (Figure 2). No cells remained in bis-acrylics and composite materials because no cells adhered to these materials. There was no significant difference between Ti alloy and milled acrylic (37.47 ± 4.99% and 31.42 ± 4.67%, respectively) (*p* = 0.22). At the same time, self-cured acrylic 1 and 2 showed 60–80% lower cell retention than milled acrylic (14.26 ± 3.76% and 7.10 ± 1.37%, respectively) (*p* < 0.0001).

### 2.3. Fibroblast Growth at Different Phases of Cell Settlement

**Figure 3:**
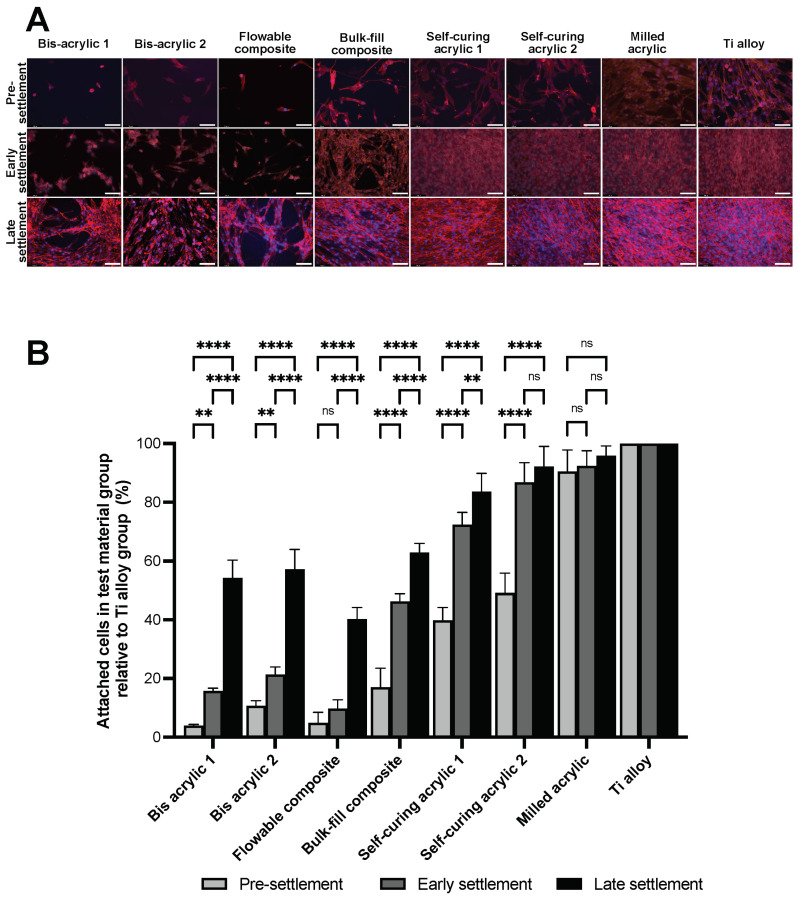
Fibroblast growth on the well surface two days after placement of test materials. (**A**) Visualization of fibroblasts: Fibroblasts were dual stained with fluorescent dyes, DAPI for nuclei, and rhodamin–phalloidin for actin filaments. Scale bars indicate 1 mm. (**B**) Percentage of the number of cells in test material groups relative to the number of cells in Ti alloy group was measured. Data shown are means ± SD. Two-way ANOVA, followed by the Fisher’s LSD post hoc test. *p* ** < 0.01, *p* **** < 0.0001. ns, Not significant.

The cells attached to the surface of the well were measured two days after the placement of a cell culture insert containing the test plate. Unlike the contact experiment, cells were observed in all groups in the proximity experiment (Figure 3A). Small cells were sparsely dispersed in the bis-acrylic and flowable composite groups until early settlement, and there were gaps between the increased cells at late settlement. The spindle-shaped cells were densely spread in the self-curing acrylic, milled acrylic, and Ti alloy group after the early settlement phase. Cell numbers were determined by counting cell nuclei, and the percentage of cells attached to the well relative to the attached cells in the Ti alloy group was calculated (Figure 3B). During the pre-settlement phase, bis-acrylic 1 and flowable composite had less than 5% of cells relative to the Ti alloy group (3.96 ± 0.40% and 4.90 ± 3.63%, respectively), while bis-acrylic 2 and bulk–fill composite had 10–20% of cells (10.72 ± 1.76% and 17.02 ± 6.50%, respectively).

The number of fibroblasts attached to the well increased from pre-settlement to the early settlement phase, except for the flowable composite group. In the early settlement phase, the percentage of self-curing acrylic 2 and milled acrylic reached above 80% (86.81 ± 6.66% and 92.42 ± 5.14%, respectively), while only 10–20% of cells relative to Ti alloy group were attached in the flowable composite group and the bis-acrylic 1 and 2 groups (9.71 ± 3.07%, 15.71 ± 1.03%, and 21.32 ± 2.61%, respectively). Even in the late settlement phase, the flowable composite was less than half of Ti alloy (40.24 ± 4.01%).

**Figure 4:**
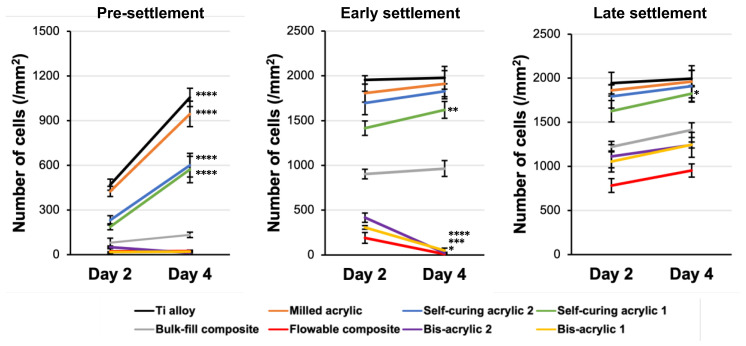
Cell propagation under three different conditions from day 2 to day 4. Data shown are means ± SD. Two-way ANOVA, followed by the Fisher’s LSD post hoc test. *p* * < 0.05, *p* ** < 0.01, *p* *** < 0.001, *p* **** < 0.0001.

Next, we evaluated cell proliferation from day 2 to day 4 (Figure 4). In the pre-settlement phase, Ti alloy, milled acrylic, and self-curing acrylic 1 and 2 groups showed significant increases, while there was no increase in the other groups. In the early settlement phase, flowable composite and bis-acrylic 1 and 2 groups showed a significant decrease. In the late settlement phases, the number of cells did not increase in flowable composite, bulk–fill composite, and bis-acrylic 1 and 2 groups, even though the cells did not reach confluence.

### 2.4. Collagen Deposition

**Figure 5:**
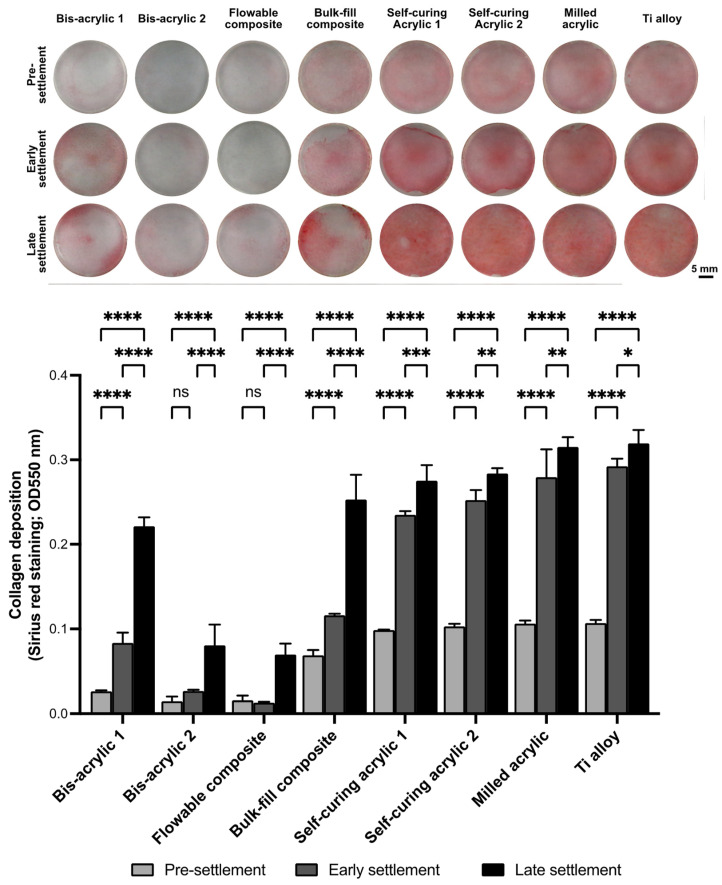
Imaging and quantification of collagen deposition using Sirius red staining two days after placement of test materials. Data shown are means ± SD. Two-way ANOVA, followed by the Fisher’s LSD post hoc test. *p* * < 0.05, *p* ** < 0.01, *p* *** < 0.001, *p* **** < 0.0001. ns, Not significant.

Finally, we evaluated the collagen deposition at three different settlement phases (Figure 5). The collagen deposition on the well was evaluated two days after the placement of the test materials. In the pre-settlement phase, the amount of collagen deposition in bis-acrylic 1 and 2 and flowable composite groups was less than one-fifth of that in the Ti alloy group. From the pre-settlement to the early settlement phase, there was no increase in bis-acrylic 2 and flowable groups. In the late settlement phase, the amount of collagen deposition remained at a low level in bis-acrylic 2 and flowable composite groups, and once exposed to these materials, there was even a reduction in the amount of collagen that was already produced. The collagen deposition in the self-curing composite and the milled acrylic group were comparable to the Ti alloy group in all phases.

## 3. Discussion

**Figure 6:**
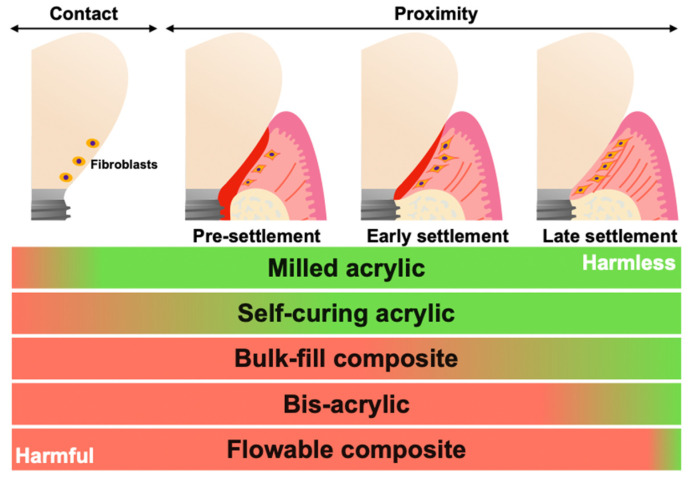
Schematic summary of results. Pre-settlement, early settlement, and late settlement mimicked the healing process of immediate implant placement with provisionals, secondary surgery for the placement of provisionals, and replacement from healing abutment to provisionals, respectively. The increased red indicated an increased level of harm, while the increased green indicated an increased level of harmlessness.

In this study, the effects of different implant provisional restorative materials on the attachment, growth, and function of human gingival fibroblasts were evaluated under different phases of cell settlement. The cells were evaluated in direct contact with the materials and in close proximity to materials. The proximity experiment was divided into pre-settlement, early settlement, and late-settlement phases. These phases mimicked the healing process of immediate implant placement with provisionals, secondary surgery for the placement of provisionals, and the replacement from healing abutment to provisionals, respectively. As a control, a biocompatible Ti alloy was used, and a total of eight different materials were tested. The results demonstrate that the harmful effects of materials on fibroblasts varied depending on the material compositions, even with the same curing modality. There was a significant difference in cell attachment, growth, or function between flowable and bulk–fill composite, between bis-acrylics, and between self-curing acrylics. In addition, we found that the harmful effects were mitigated when materials were in proximity to cells compared to direct contact. However, the harmful effects of bis-acrylics and flowable composite persisted even in the late settlement phase. Cytotoxicity of these materials would exceed the damage tolerance of fibroblasts. The summary of these results is shown in Figure 6. To our knowledge, this is the first study to evaluate the influence of provisional materials on cells with different phases of cell settlement.

Some studies have shown that material compositions such as monomers, polymerization initiators, and filler particles influence their cytotoxicity [48,49,50,51,52,53,54]. It is known that unreacted monomers exert critical biological effects on cells [15,55,56]. Bis-GMA, a main component of bulk–fill and flowable composites, is released at high levels even 28 days after polymerization [57]. UDMA, a main component of bulk–fill composite, is also persistently released, and bis-GMA is eluted at higher concentrations than UDMA [35,58]. Results on cell attachment and proliferation being the highest in the milled acrylic group suggest that there is lower residual monomer elution from milled acrylic [28]. Our results suggest that bis-GMA and UDMA are more toxic than MMA, while UDMA is less toxic than bis-GMA.

Polymerization initiators such as benzoyl peroxide (BPO) and camphorquinone (CQ) compromise cell viability [47,59,60,61]. BPO, a major initiator for self-curing acrylic resin, is broken down during polymerization to release radicals that injure surrounding cells [52,53,54]. CQ is well known as a photoinitiator for light-curing acrylic and composite resin, and it produces a pair of free radicals through proton abstraction [62,63]. In our study, self-curing acrylic was less cytotoxic than flowable and bulk–fill composites. This suggests that BPO is likely to be less cytotoxic than CQ. Milled acrylic is made of PMMA disks molded under high temperature and pressure in an anhydrous environment, and it has superior mechanical properties to conventional heat-polymerizing acrylics [64]. In addition, milled acrylics have favorable cytocompatibility due to lower residual monomer composition than self-cured acrylics [29], which was confirmed in the present results. The number of attached cells was different within self-curing acrylic groups in the contact experiment. The major difference between self-curing acrylics is diethyl phthalate as a plasticizer. Diethyl phthalate is leached from the acrylic materials, causing cellular damage by producing reactive oxygen species [65]. Self-curing acrylic 1 contains 10–20% diethyl phthalate as a composition of the powder. Thus, self-curing acrylic 1 is thought to be more harmful than self-curing acrylic 2.

After the early settlement phase, cell proliferation was not observed in self-curing acrylic 2, milled acrylic, and Ti alloy groups. Considering their low cytotoxicity and the time from cell seeding to the material placement, it was thought that the number of cells already reached a plateau two days after the placement of the materials. On the other hand, self-curing acrylic 1 slightly impaired cell growth, so there was capacity for cell propagation through to the late settlement phase. In bis-acrylics and flowable composite groups, only a few cells could survive in pre-settlement exposure. In the early settlement phase, the deleterious effect was still at a high level and resulted in a decreased number of cells. Even in the late settlement, the negative effect persisted. It would be explained that TEGDMA has various effects on cells, not only causing apoptosis but also inhibiting cell proliferation or differentiation [66,67]. The reduced cytotoxicity of bulk–fill composite compared to flowable composite may be partially attributed to the lower concentration of TEGDMA. In addition, the wettability and roughness of the materials’ surface affect the initial cellular behavior [68,69,70,71,72,73]. Cell attachment and proliferation are more favorable on a hydrophilic surface compared to a hydrophobic surface. Additionally, the smooth surface is more favorable compared to the rough surface. SEM images in a previous study showed that the surface roughness differed among test materials [46]. Milled acrylic and Ti alloy have a smooth surface; on the other hand, bis-acrylic, composite, and self-curing acrylics have a rough surface. Therefore, not only the compositions of materials but also the surface topography may affect cytocompatibility.

The soft tissue seal at the abutment and prosthetic material interface, if established, may play an important role in preventing peri-implantitis. Therefore, cell adhesion or cell retention by the material is an essential factor in determining the cytocompatibility of provisional implant materials. Of note, the milled acrylic retained a comparable number of cells to Ti alloy. Considering that cell retention was significantly compromised on self-curing acrylics, this result represents an additional benefit favoring milled acrylic. Increased cell attachment and proliferation do not necessarily result in increased cell retention. However, as vividly observed in the fluorescence microscopy images, the increased cell density, cell spreading, and cytoskeletal development thus improved intercellular adhesion and may have increased cell retention on milled acrylic. Although this study used a chemical detachment protocol, other methods of detachment, such as mechanical and vibrational detachment or their combination with chemical detachment, are an area of interest since the provisional material–fibroblast interface may be subjected to micro-movements in vivo.

In the proximity experiment, a cell culture insert containing each test plate was placed in a well where fibroblasts were pre-cultured. As expected, the damage to the fibroblasts was reduced compared to the contact experiment. In all test groups, as the number of settled cells before placement of the insert increased, the harmful effect of materials decreased. Cell proliferation from day 2 to day 4 indicated that Ti alloy, milled acrylic, self-curing acrylics, and bulk–fill composite showed an increase in the pre-settlement phase, whereas bis-acrylics and flowable composite decreased. Of note, bis-acrylics and flowable composite had almost no cells remaining. A similar trend was also observed in the early settlement phase. Therefore, the cytotoxicity of these materials might exceed the tolerance of fibroblasts without direct contact.

Regarding collagen deposition, in the early and late phases, collagen production was thought to be comparable among all groups before material placement. Thus, the amount of collagen was not influenced by the materials, although the number of cells decreased. However, the collagen deposition in bis-acrylic 2 and flowable composite was limited. Not only was there the inability for new collagen production from the reduced number of cells, but also the damaged fibroblasts were secreting matrix metalloproteinases (MMPs), resulting in decreased deposition [74,75,76]. Bis-acrylic 1 exhibited two times more collagen deposition compared to bis-acrylic 2, while bulk–fill composite showed three to five times more collagen deposition than flowable composite. Considering these results, the difference between materials may not only be due to the decrease mediated by fibroblast-produced collagenases but also to the inherent capacity of certain materials to degrade collagen. BisGMA and UDMA do not degrade collagen, but other proprietary components may possess collagen-degrading properties. Further studies are needed with a more comprehensive analysis to determine whether the properties of provisional materials degrade collagen or not.

Due to the chemistry of the resinous/polymerizing materials, we believe that the standardized preparation of materials, in particular the storage time, directly affects the results. The materials elute residual monomer after initial polymerization [15,18,39]. Although the amount of residual monomer reaches its peak within 24 h, the peak differs depending on the materials. Considering the number and types of materials examined, conducting the experiments immediately after materials fabrication and comparing the peaks is extremely difficult, especially because these fibroblasts are so susceptible when isolated from the host response. Therefore, we standardized the storage time of all test materials for two weeks under standardized conditions prior to proceeding with the experiments. Although the concentration decreases after the peak, there is a sustained release of the elutes that impacts the cellular interactions. We are fully aware that materials with different storage times need to be tested in future studies.

This study focused on the effect of the materials on the initial activity of fibroblasts under different conditions. The initial cellular response and reaction are critically important to subsequent initial site healing, cellular function, and long-term tissue health. Indeed, if cells are exposed to cytotoxins, they undergo rapid cell death and cannot function properly. This study reveals a great variation in the initial fibroblast activities in various environments in response to different provisional materials. However, from a clinical perspective, it is necessary to examine whether the materials cause inflammation in the peri-implant tissues. In order to assess inflammatory reactions in peri-implant tissue, in vivo studies should be conducted. In vivo studies enable the investigation of site-specific inflammatory cytokine production and the infiltration of inflammatory cells within the tissues. Such studies will contribute to a more comprehensive understanding of the clinical use of provisional materials.

Peri-implant tissue is composed of junctional epithelium and connective tissue, consisting of keratinocytes and fibroblasts, respectively. In this study, we focused on the soft tissue seal around the most apical portion of provisional restorations, in the closest proximity to the crestal bone, and established an experimental model to mimic this relationship by investigating the effects on fibroblasts. The rationale behind this focus is that if the connective tissue breaks, inflammation will rapidly spread to the alveolar bone, leading to bone resorption. However, the epithelium also plays an important role in establishing peri-implant soft tissue seals as keratinocytes adhere to titanium through hemidesmosomes. If the hemidesmosomes formed onto low-cytotoxic provisional materials, it potentially contributes to better cell retention. Further experiments using keratinocytes and the derivatives of epithelial cells were desired. Future studies other than those already mentioned may include the development of materials with high cytocompatibility properties (i.e., N-acetyl cysteine and tri-n-butyl borane) to improve reduction–oxidation systems and reduce radical production [53,61,77,78].

## 4. Materials and Methods

### 4.1. Material Preparation

**Figure 7:**
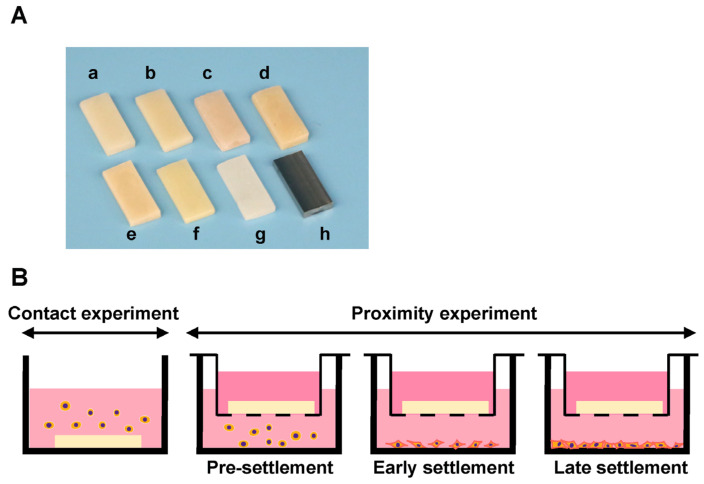
Test materials and experimental design for counting cells. (**A**) Rectangular plates (6 mm × 14 mm, 2 mm thick) were prepared. a, bis-acrylic 1; b, bis-acrylic 2; c, flowable composite; d, bulk–fill composite; e, self-curing acrylic 1; f, self-curing acrylic 2; g, milled acrylic; and h, Ti alloy. (**B**) Attached fibroblasts were counted to determine the effect of test materials under various cell conditions. The experiment was divided into contact experiments and proximity experiments. The contact experiment was the quantification of fibroblasts directly attached to test material. The proximity experiment was divided into three phases: pre-settlement, early settlement, and late settlement. In the proximity experiment, the quantification of fibroblasts attached to the well of the culture dish (20 mm diameter) was conducted immediately, 24 h or 72 h after the placement of a cell culture insert containing each test material.

Eight different specimens were fabricated in a rectangular plate form (6 mm × 14 mm, 2 mm thickness) for evaluation. The prepared test plates and their principal compositions are shown in Table 1. Two bis-acrylics, flowable and bulk–fill composites, and two self-curing acrylics were prepared using standardized silicone molds and according to the manufacturer’s instructions (Figure 7A). Three plates of each material were prepared for each experiment. A light curing device (Coltolux LED; Coltène, Altstätten, Switzerland) was used to polymerize the light curing materials with a wavelength of 450–470 nm and an intensity of 1275 mW/cm^2^ for 30 s. Milled acrylic plates were designed using CAD software (123D^®^ Design version 2.2.14, Autodesk, Inc., San Rafael, CA, USA), and manufactured from PMMA disks with a milling machine (Versamill 5 × 200, Axsys Dental Solutions, Wixom, MI, USA) using CAM software (HyperDENT^®^ version 9.0.2, Synergy Health, Sydney, Australia). Two weeks after preparation, all acrylic plates were washed with a steam cleaner and disinfected with 75% ethanol. Machined Ti alloy plates were also manufactured as a positive control. No surface polishing was performed to milled PMMA and Ti alloy.

### 4.2. Cell Culture and Material Placement

Human gingival fibroblasts were obtained from ScienCell Research Laboratories (Carlsbad, CA, USA) and grown in a fibroblast medium supplemented with 5% fetal bovine serum (FBS), 1% Fibroblast Growth Supplement-2, and 1% penicillin/streptomycin solution. At 80% confluence, the cells were detached using 0.05% trypsin-EDTA solution and seeded at density of 4 × 10^4^ cells/well. To evaluate the influence of materials on cells in various conditions, seeding process was divided into two experiments: the cells in direct contact with test materials (contact experiment) and in close proximity to test materials (proximity experiment). The proximity experiment was further divided into three phases: pre-settlement, early settlement, and late settlement phase (Figure 7B). In the contact experiment, cells were seeded onto each test material placed in a well (20 mm diameter) of 12-well culture plates. In the proximity experiment, cells were seeded onto each well without materials. For evaluation of the pre-settlement phase, a cell culture insert with a 0.4 µm pore size containing the test plate was placed into a well of 12-well immediately after cell seeding. In the early settlement phase, the test plates were placed at 24 h after cell seeding. In the late settlement phase, the plates were placed at 72 h after cell seeding. The culture medium was renewed every three days. The UCLA Institutional Biosafety Committee (BUA-2-22-036-001) approved the study protocol.

### 4.3. Quantification of Attached and Propagated Cells with Fluorescence Microscopy

The number of attached fibroblasts was counted to determine the effect of test materials under various culture conditions. The contact experiment referred to the quantification of fibroblasts directly attached to test materials, while the proximity experiment referred to the quantification of fibroblasts attached to the well after placement of the cell culture insert containing the test material. In the contact experiment, two and four days after cell seeding, cells on the test plates were fixed in 10% formalin, permeabilized with 0.5% Triton X-100, and blocked with 1% BSA. Subsequently, the cells were dual stained with fluorescent dyes: 4′,6-diamidino-2-phenylindole (DAPI) to identify nuclei and rhodamine–phalloidin for actin filaments and observed with fluorescence microscopy (DMI6000B, Leica Microsystems, Wetzlar, Germany). In the proximity experiment, two and four days after incubating the cells with the test materials, the attached cells on the wells were fixed and stained. The number of cells was quantified by counting the cell nuclei in the taken images (Image J version 1.53, NIH, Bethesda, MD, USA). Increases in the number of cells from day 2 to day 4 were measured as cell proliferation.

### 4.4. Cell Retention Assay

The retention of fibroblasts attached to each test plate was evaluated by calculating the percentage of remaining cells after chemical detachment, as reported previously [79]. Four days after seeding, to remove any extra cells not attached to the samples, carefully transfer and dip the samples into a new well containing PBS. After removing the PBS completely by aspiration, 0.0125% trypsin/EDTA was added to detach the attached cells, and the samples were incubated at 37 °C for 2 min. A hematocytometer was used to count the number of detached cells. The remaining cells on the material surface were completely detached with 0.0125% trypsin-EDTA at 37 °C for 10 min, and completely detached cells were counted. The percentage of remaining cells was calculated using the following formula:

Percentage of remaining cells (%) = {(Number of completely detached cells at 10 min − Number of detached cells at 2 min)/(Number of completely detached cells at 10 min)} × 100

### 4.5. Collagen Deposition

Collagen deposition produced by fibroblasts in the proximity experiment was measured two days after placement of inserts containing test plates by picrosirius red staining (Polysciences Inc., Warrington, PA, USA). The cells were washed with PBS and fixed in 10% formaldehyde for 10 min. Subsequently, the collagen fibers were stained with 0.1% picrosirius red solution for 60 min at room temperature, after which 0.1 N sodium hydroxide was added for 60 min to elute the bound dye. The supernatant was measured at an absorbance of 550 nm using a microplate reader.

### 4.6. Statistical Analysis

Results are expressed as means ± standard deviations (SD). All experiments were performed in triplicate (*n* = 3). The eight materials were compared by one-way analysis of variance (ANOVA) followed by the Tukey–Kramer post hoc test. Furthermore, two-way ANOVA followed by post hoc comparisons using Fisher’s LSD test was performed to evaluate the changes between test materials at different time points. *p*-values less than 0.05 were deemed statistically significant.

## 5. Conclusions

This study indicated that the harmful effects of provisional materials on gingival fibroblasts vary depending on the curing modality and material composition. Pre-settlement of cells mitigated the harmful effects, implying the susceptibility to material toxicity varies depending on the progress of wound healing and tissue condition. However, cell pre-settlement was not sufficient to fully restore the fibroblastic function to the normal level. Notably, bis-acrylics and flowable composites still exhibited considerable adverse effects even in the late-settlement phase, whereas milled and self-curing acrylics demonstrated excellent and acceptable biocompatibility when compared to other materials. These results provide valuable information for clinical practice to optimize peri-implant health and enhance material cytocompatibility in future developments of provisional materials.

## Figures and Tables

**Table 1:** Materials used in this study.

Materials	Principal Compositions	Curing Modality
Bis-acrylic 1 (Integrity^®^ Multi + Cure Temporary Crown and Bridge Material, Dentsply Sirona, Chariotte, NC, USA)	Acrylates and methacrylates (bis- and multifunctional) Barium boro alumino silicate glass	Dual-curing (chemical-curing and light-curing)
Bis-acrylic 2 (Visalys^®^ Temp, Kettenbach GmbH & Co. KG, Eschenburg, Germany)	Aliphatic dimethacrylate, Poly(alkyleneglycol) diacrylate, hydroquinone monomethyl ether	Chemical-curing
Flowable composite (Aeliteflo™, BISCO Inc., Schaumburg, IL, USA)	Bis-GMA, TEGDMA	Light-curing
Bulk–fill composite (Aelite™ Aesthetic Enamel, BISCO Inc.)	Ytterbium Fluoride, Bis-GMA, UDMA Bis-EMA, TEGDMA	Light-curing
Self-curing acrylic 1 (JET Tooth Shade, Lang Dental Manufacturing Company Inc., Wheeling, IL, USA)	(liquid) MMA, N,N-Dimethyl-p-Toluidine (powder) 2-Propenoic acid, 2-methyl-, methyl ester homopolymer, Diethyl Phthalate	Chemical-curing
Self-curing acrylic 2 (UNIFAST™ Trad, GC, Tokyo, Japan)	(liquid) MMA, N,N-dimethyl-p-toluidine (powder) PMMA, Dibenzoyl peroxide	Chemical-curing
Milled acrylic (Vivid PMMA Disc, Pearson™ Dental Supply Co., Sylmar, CA, USA)	PMMA	Pre-curing (chemical-curing with high pressure and high temperature)
Ti alloy	Ti-6Al-4V (Grade 5)	-

Abbreviations: UDMA, urethane dimethacrylate; Bis-EMA, bisphenol A Ethoxylate Dimethacrylate; TEGDMA, triethylene glycol dimethacrylate; Bis-GMA, bisphenol A glycidyl methacrylate; MMA, methyl methacrylate; PMMA, poly(methyl methacrylate).

## Data Availability

The data are available upon request from the corresponding author.
